# Perinatal mental health care goes digital: Opportunities for digital health to revolutionize the care delivery for healthier pregnancies and outcomes

**DOI:** 10.1371/journal.pdig.0000773

**Published:** 2025-04-01

**Authors:** Bindu Chanagala, Suneet P. Chauhan, Sheehan D. Fisher, Matthew Hoffman, Martin G. Frasch

**Affiliations:** 1 nurtur health Inc, Dover, New Hampshire, United States of America; 2 Department of Obstetrics & Gynecology, Center for Women & Children’s Health Research, ChristianaCare, Wilmington, Delaware, United States of America; 3 Department of Psychiatry & Behavioral Sciences, Feinberg School of Medicine, Northwestern University, Chicago, Illinois, United States of America; 4 Department of Obstetrics & Gynecology, Institute on Human Development and Disability, University of Washington, Seattle, Washington, United States of America; Iran University of Medical Sciences, IRAN, ISLAMIC REPUBLIC OF

## Introduction

Perinatal mental health care has remained on the sidelines of the maternal health crisis in the United States (US) for too long [1,2]. This team of authors gathered a consortium to contribute to a tidal wave of efforts aiming to remove that blind spot, or, one might even argue, a tabu existing in many societies around the world, not just in the US.

There is no better way to lead such an effort than by lending a voice to a lived experience. We are fortunate that one of our co-authors and team members has such a first-hand story to share, not only of the suffering from depression but also an extraordinary effort to solve the underlying problem. Why extraordinary? Because even now, women founders encounter stigma and biased perceptions of their abilities to lead, build, and grow amazing teams and companies, as if, opening up about their lived experiences and doing something about them for all expectant mothers were not enough.

Consequently, breaking somewhat with the traditional scientific style of plural narrative and reasoning throughout, we partitioned this article by lending first this one voice its full power by speaking from herself, i.e., sharing a first-person account of this experience in Parts 1 and 2. We then return to the outline of the field of post-partum depression, the most common form of perinatal mental morbidity, and ways in which digital health technologies are uniquely suited to overcome the existing shortcomings in care (Part 3).

### Part 1. Lived experience through one voice, but a story of many

Growing up in India, mental health, much less postpartum depression, was rarely mentioned. The pressure to excel academically was so intense that there was little time to even consider whether I was mentally okay. Competing with thousands of students left no room for self-reflection or mental well-being. When my husband and I moved to the US in 2008 to pursue our Master’s degrees, I continued to hustle, juggling a full-time job with evening classes. As a young bride, far from family, I was accustomed to being busy and pushing through challenges.

When I became pregnant with my first child, everyone around me—both near and far—was filled with excitement. I was thrilled to start a family with my husband, whom I love dearly. Throughout the pregnancy, I continued working full-time while navigating the complexities of US healthcare as an engineer working for an integrated healthcare system. My first disappointment came when I gained a significant amount of weight, and I often wished my mother was there to comfort me and help with the preparations of being a mother. Without family support, dealing with the changes in my body, and then the shock of a Cesarian delivery, I was overwhelmed.

While meeting my beautiful baby girl was a joyous moment, it wasn’t long before the dark days and nights set in.

I didn’t understand what I was feeling. As a young immigrant mom, I was hesitant to give voice to my emotions, fearing the possibility of having my baby taken away due to my lack of understanding of the legal system in the US. I had never heard of postpartum depression, let alone considered discussing or learning about it.

One of the darkest nights of my life was when my precious daughter was crying uncontrollably in the middle of the night. I was utterly exhausted, both mentally and physically, and felt completely drained of energy. In that moment of despair, a terrifying thought crossed my mind—I considered dropping her on the floor.

The thought horrified me. It was a fleeting thought.

Instead of acting on it, I dropped her in the middle of the bed, hoping to get my husband’s attention. A testament to his resilience and compassion, he immediately took over and gave me a much-needed break. But the guilt, sadness, and shame of that moment lingered. I couldn’t understand why I would even think of doing such a thing to my innocent, precious baby. She is a gorgeous 14-year-old now, yet that memory remains vivid, a moment I wish I could erase.

For years, that incident haunted me. Despite living in what is considered the greatest nation in the world, I saw how the system failed mothers like me. I knew I wanted to address these issues, but life kept moving forward. I had two more beautiful daughters, and I discovered that sunlight helped calm my mind during the postpartum period. However, everything fell apart when I lost my fourth baby, my sweet boy, nearly halfway through the pregnancy. Nothing could have prepared me for that experience, and the depression that followed was suffocating. The hospital staff were compassionate and supportive, but in the weeks, months, and years that followed, I struggled alone, lacking support. I felt like a mess, my brain fogged and incapable of even simple social interactions, let alone leading a team of brilliant engineers at work. But I fought through it because I needed to. My career, which I had worked so hard to build, and the needs of my family kept me going.

In 2022, a dream came true—I was accepted into the MBA program at MIT Sloan School of Management. It was a personal challenge, a chance to reinvent myself and rethink the second half of my career. Halfway through the program, a cohort fellow asked me what I wanted to do with this degree. That’s when I opened up about my struggle with postpartum depression and the loss of my fourth baby. She suggested that I connect with Kristen who by then was already working on this problem, and that’s how I met my co-founder. Together, we gave birth to nurtur at MIT. We began winning competitions for our idea to predict postpartum depression coupled with a digital therapeutic and were accepted into MIT’s prestigious accelerator, delta v, where we started conducting market research on this critical issue.

### Part 2. From lived experience of postpartum depression to solving a global mental health challenge

In India, where mental health was rarely discussed, my journey has profoundly shaped my perspective as a co-founder and Chief Product Officer of nurtur, particularly in the field of digital health innovation. My lived experiences navigating the mental health challenges of pregnancy, postpartum depression, and the devastating loss of a pregnancy illuminated the glaring gaps in maternal mental health care.

As a co-founder of nurtur, my leadership expertise has been pivotal in transforming these personal insights into actionable solutions. The development of our digital cognitive behavioral therapy platform exemplifies how innovation, driven by both data and empathy, can bridge systemic gaps in care delivery. By combining cutting-edge technology, evidence-based interventions, and a commitment to preventive care, our work aligns deeply with the broader themes of my writing: empowering women, addressing health inequities, and leveraging digital health to create scalable, impactful solutions for maternal mental health.

### Part 3. Team experience

The shared personal lived experience underscores the broader implications of the impact that the continued neglect of perinatal mental health has had on women’s and children’s health, as well as the well-being of families as a whole. The statistics are staggering in the US, for example, one in five women experience postpartum depression, yet 85% go undiagnosed and untreated [1,2]. A failure to recognize that untreated postpartum depression significantly impacts both mother and child [3–6]. The prevalence of postpartum depression has increased by 105% in the last decade alone [7].

The situation globally is no less concerning.

The prevalence of postpartum depression in India is overall similar to the US and also shows major geographic variations with Northern regions averaging 15% while Southern regions average 26% [8–10].

More generally in low- and middle-income countries (LMICs), the impact of digital health strategies on improving antepartum, perinatal, and postnatal health is seen favorably in various studies. Digital health strategies have evolved to improve maternal survival and well-being in LMICs. There is evidence of mobile health (mHealth) interventions in bridging healthcare gaps, promoting equitable access to maternal health services, and improving health outcomes [11,12]. Especially during care delivery disruptions, as happened during the COVID-19 pandemic, mHealth technologies have demonstrated the ability to maintain and enhance the standard of care [13]. The importance of ensuring the cost-effectiveness and sustainability of digital health services has been highlighted [14]. That is true anywhere, not just in LMICs if these technologies are to scale successfully with massive adoption and positive impact on health outcomes. The potential of the impact of digital health technologies, especially those leveraging AI, in resource-constrained environments, especially, but not limited to LMICs, has been emphasized [15].

Despite the many research studies, practical solutions capable of improving lives are still lacking. Why isn’t more being done about this? Around the same time, we heard about the tragic loss of a mother, who died by suicide just nine days after delivering her baby in Massachusetts, sadly, one of many. This fueled our mission to address the gaps in mental healthcare. During our research, we connected with Dr. Martin Frasch, whose recent paper identified nine pre-pregnancy factors that could predict postpartum depression [16]. Through our platform, we can increase screening rates [16], with women completing the Edinburgh Postnatal Depression Scale every 2 weeks from the time their baby is born for up to 12 months. This allows us to catch cases early and ensure timely human intervention for those who need it most.

But we didn’t stop there.

We discovered that postpartum depression can be prevented by up to 53% using a validated pregnancy-specific protocol of self-directed interpersonal therapy known as the ROSE program [17], yet this intervention is not widely discussed or implemented. So, we developed a self-directed digital cognitive behavioral therapy program, where high-risk women can complete eight sessions during pregnancy and two more postpartum.

### Part 4. Preliminary findings

In the following, we share insights from the early deployment of the nurtur digital perinatal mental health platform. Out of the 52 users invited to the nurtur platform, 32 (61.5%) enrolled. While this demonstrates strong initial engagement, adherence in terms of completion rates reveals room for improvement compared to the industry standard of 40% adherence typically seen in digital therapeutics [18].

Among the onboarded users, 6 (11.5% of all invites and 18.8% of active users) completed all 8 sessions, achieving full adherence. Meanwhile, 2 users (3.8% of all invites) completed half the program, 3 (5.8%) completed 2 sessions, and 4 (7.7%) completed at least 1 session. An additional 3 users (5.8%) only partially completed a session, while 10 users (19.2%) created accounts but did not initiate any sessions.

When compared to the 40% industry benchmark for full program completion, nurtur’s completion rate of 11.5% highlights a gap that needs to be addressed to drive higher engagement. So far, the present performance has been supported by proactive nudges via email and text messages, which played a crucial role in motivating users to progress through the program. To bridge the gap and boost completion rates, further research and development are necessary to determine the optimal frequency, tone, and timing of these nudges as well as to identify additional mechanisms of adherence boosting. By refining these strategies, nurtur will improve completion rates to align with or even exceed industry standards while maintaining its already strong onboarding/enrollment performance.

### Part 5. Outlook

Digital health-powered companies like nurtur are on a mission to fill the gaps in maternal mental health care and make sure no mother has to suffer in silence and that every mother has the opportunity to see their child grow. Why are we confident we will succeed?

We live in the era of convergence of the crucial ingredients necessary for a transformation like this to take hold and grow.

First, there has been an unprecedented uptake in digital health technologies for wellness and telemedicine, including those powered by artificial intelligence and machine learning (AI/ML) [19,20].

Second, access to these technologies has been improving [20].

Third, there has been a growing need to utilize more privacy-conscious, individualized approaches to mental health care in ways that do not stigmatize [21].

Fourth, digital interventions overcome geographical barriers. This advantage addresses the growing shortage of mental health professionals in at least 30% of expectant mothers in the US live in maternity care deserts [22,23].

Fifth, digital health interventions, particularly in mental health, reduce the cost of care to both the mother and the payor while improving personalization, which also aids in making the care more culturally sensitive and inclusive. The added interactivity of care also contributes to improved adherence and reduced attrition rates increasing the overall effectiveness [24].

Sixth, digital health interventions are simultaneously scalable [25].

Challenges in the implementation of digital health intervention remain to be overcome:

1) While access to digital technology has improved, its dependence on the Internet poses limitations in areas where the Internet access itself is limited - this number worldwide still represents 32% of the global population but has been declining steadily [26];
2) Privacy concerns.
3) Lack of unified definitions for user engagement and adherence [27];
4) In PPD screening in particular, limited availability of ML models has been noted that are culturally sensitive, i.e., specifically control for ethnic or racial aspects which act as proxy for different access to or engagement with mental health care [28]. This requires a thoughtful approach to the selection of predictor variables for model training as well as in the choice of the response variable. The authors recommend using psychometric screeners rather than, for example, ICD-10 codes or pharmacy records, due to the inherent bias encoded in these instruments of measuring the outcome.
 For example, if the model requires information about race-dependent behavior, then such a predictor variable would naturally be biasing the model’s prediction. This way of thinking requires a causal approach to defining the variables for which a causal inference framework could be recommended as a formal methodology. In some instances, e.g., when querying about access to mental health care, an important variable to assess, it is also important to recognize that it may reflect underlying inequity issues that reflect systemic racism or barriers in access to care. From an ML standpoint, such variables can still be powerful components of a predictive model. Access to medication for mental health can be a similar example. Therefore, making the ML model behavior as transparent as possible to the users, providers or patients is important.

There is today an exciting opportunity for a massive global impact on health outcomes through digital health technologies, especially in mental health. This is evidenced by the many studies to date that highlight shared fundamental health care problems around the world, regardless of the socioeconomic situation: resource-constrained environments reducing access to care paired with significant advances in access to the Internet around the world make digital health a prime technology to tackle local and global acute and chronic health problems at an unprecedented scale. However, while the opportunity is global, the mHealth solutions need to be built with each specific health facet and population group in mind to ensure that the AI/ML performance does not deteriorate or bias over time or in certain groups of individuals.
